# Differential in Gel Electrophoresis (DIGE) Comparative Proteomic Analysis of Macrophages Cell Cultures in Response to Perthamide C Treatment

**DOI:** 10.3390/md11041288

**Published:** 2013-04-17

**Authors:** Annalisa Vilasi, Maria Chiara Monti, Alessandra Tosco, Simona De Marino, Luigi Margarucci, Raffaele Riccio, Agostino Casapullo

**Affiliations:** 1 Department of Pharmacy, University of Salerno, Via Ponte don Melillo, 84084 Fisciano (SA), Italy; E-Mails: a.vilasi@ibp.cnr.it (A.V.); tosco@unisa.it (A.T.); lmargarucci@unisa.it (L.M.); riccio@unisa.it (R.R.); 2 Department of Pharmacy, University of Naples, Via D. Montesano 49, 80131 Naples, Italy; E-Mail: simona.demarino@unina.it

**Keywords:** marine sponge peptides, differential in gel electrophoresis, chaperones, apoptosis, inflammation

## Abstract

Secondary metabolites contained in marine organisms disclose diverse pharmacological activities, due to their intrinsic ability to recognize bio-macromolecules, which alter their expression and modulate their function. Thus, the identification of the cellular pathways affected by marine natural products is crucial to provide important functional information concerning their mechanism of action at the molecular level. Perthamide C, a marine sponge metabolite isolated from the polar extracts of *Theonella swinhoei* and endowed with a broad and interesting anti-inflammatory profile, was found in a previous study to specifically interact with heat shock protein-90 and glucose regulated protein-94, also disclosing the ability to reduce cisplatin-mediated apoptosis. In this paper, we evaluated the effect of this compound on the whole proteome of murine macrophages cells by two-dimensional DIGE proteomics. Thirty-three spots were found to be altered in expression by at least 1.6-fold and 29 proteins were identified by LC ESI-Q/TOF-MS. These proteins are involved in different processes, such as metabolism, structural stability, protein folding assistance and gene expression. Among them, perthamide C modulates the expression of several chaperones implicated in the folding of proteins correlated to apoptosis, such as Hsp90 and T-complexes, and in this context our data shed more light on the cellular effects and pathways altered by this marine cyclo-peptide.

## 1. Introduction

Marine ecosystem represents an immense source of chemical entities. Secondary metabolites produced by marine organisms, especially sponges, are the result of millions of years of evolution and natural selection. They have an intrinsic ability to modulate biological processes by interacting with proteins involved in different physiological and pathogenic pathways [1]. Indeed, several marine derived drugs are approaching phase II/III clinical trials for the treatment of cancer, analgesia, allergy, and cognitive and inflammatory diseases [2,3]. 

In this scenario, the identification of the cellular pathways in which marine natural products are involved is crucial to provide important functional information, in order to understand their effects and unravel their molecular mechanism of action [4,5,6]. Nowadays, this issue can be achieved by means of mass spectrometry-based analytical techniques, which are also developing in the marine research area. Marine proteomics is a rapidly expanding field aimed to assess the molecular profiling of marine fauna, such as microbes, plants, invertebrates, and vertebrates [7,8,9,10]. 

**Figure 1:**
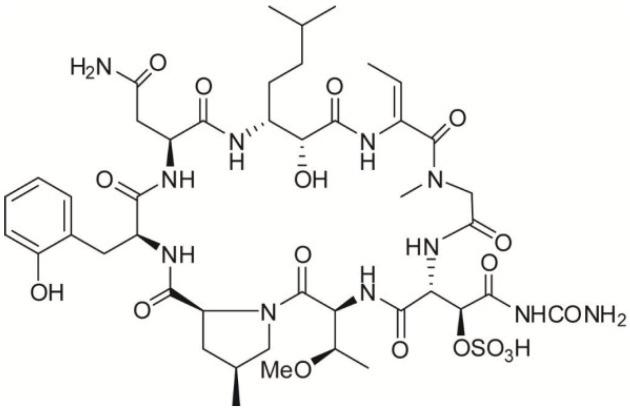
Structure of Perthamide C.

Marine organisms of the genus *Theonella* are an excellent source of bioactive peptides, endowed with interesting biological activities [11,12,13]. Among them, perthamide C (Figure 1) is a novel cyclic octapeptide isolated from the polar extract of *Theonella swinhoei* [14]. The most striking structural feature of perthamide C is the prevalence of non-ribosomal amino acids, suggestive of a possible symbiotic microorganism origin for this metabolite. 

Indeed, perthamide C is characterized by an unprecedented 25-membered macrocycle skeleton including eight aminoacid residues, seven of which—γ-methylproline, *N*-methylglycine, *N*-δ-carbamoyl-β-OSO_3_-asparagine, *o*-tyrosine, 2-aminobutenoic residue (dABU), *o*-methylthreonine, and the β-amino acid AHMHA (3-amino-2-hydroxy-6-methylheptanoic acid)—are non ribosomial [14].

Perthamide C showed in *in vivo* preliminary test remarkable anti-inflammatory properties, since it reduced in a dose-dependent fashion the carrageenan-induced paw oedema in mouse, and promoted TNF-α down-regulation and IL-8 release in primary human keratinocyte cell lines [14]. More recently, perthamide C was discovered to selectively interfere with the NO release triggered by eNOS or iNOS without affecting COX-1 or COX-2, altering the inflammatory response through a reduction of vascular permeability, neutrophile infiltration and lymphocyte proliferation [15]. Moreover, in a recent study, we identified the molecular chaperones heat shock protein-90 (Hsp90) and glucose regulated protein-94 (GRP-94) as perthamide C prominent biological targets by a chemical proteomic approach [16]. Perthamide C was also found to modulate the pro-apoptotic activity of cisplatin, in liver hepato-carcinoma cell lines (HepG2) [16]. 

On the basis of these evidences, we decided to investigate the effects of perthamide C in a living cell system, measuring the changes induced by the natural product on the proteins expression levels. In particular, we have evaluated the changes in the protein expression of murine macrophage cells exposed to perthamide C, by means of two-dimensional differential in gel electrophoresis (DIGE) and mass spectrometry. 

## 2. Results and Discussion

A two-dimensional DIGE proteomic approach was applied to measure the proteome changes of macrophage cells (J774.A1) in response to the treatment with perthamide C. The cells were grown in normal conditions and then treated with 10 μM perthamide C for 24 h. The soluble proteins recovered after cell lysis were labeled using the CyDyes DIGE Fluors, a set of three different fluorescent dyes specifically designed for detecting protein abundance differences. Briefly, an equal amount of the protein samples coming from control and perthamide C-treated cells was covalently labeled with the Cy3 and Cy5 dyes, respectively, and then the samples were mixed in 1:1 ratio and loaded on a 2D gel system. The Cy2 dye was used to label the internal standard, obtained by mixing an equal amount of all samples, allowing a significant quantitative comparison of proteomic variations. 

**Figure 2:**
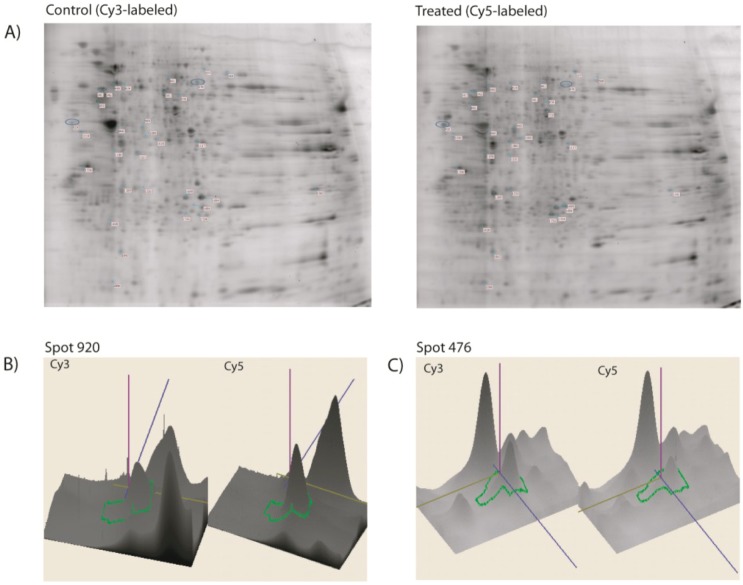
(**A**) Representative two-dimensional differential in gel electrophoresis (2D-DIGE) images of control and perthamide C-treated samples, depicting a rich array of fluorescent spots. Equal amounts of Cy2 (standard with equally mixed samples), Cy3 (Control), and Cy5 (perthamide C treated) labeled samples were mixed and then separated on analytical 2D-DIGE; (**B** and **C**) DIGE quantification: three-dimensional view of the control and drug-treated spots (spots 920 and 476 on b and c panels, respectively) shows significant differences in protein expression. The spot intensities and the relative expression ratio were computed using the ImageMaster 2-D Platinum dedicated software.

A total of six gels were run to achieve a statistically significant measure of the differences in protein expression between the control and the perthamide C-treated samples. In the following software analysis, more than 1000 proteins spots were detected on each CyDye-labeled gel, in the pH range 3–10. All protein spots were then quantified, normalized and inter-gel matched. Based on a quantitative image analysis, 33 protein spots showed a significant difference in intensity when compared with the control sample. Figure 2 shows a representative 2-D DIGE protein map of the perthamide C-treated and control samples along with three-dimensional view examples of spot intensities. The protein expression changes were considered significant only when their values exceeded the threshold settings (fold change ≥1.6, *p* < 0.05). Among the differentially expressed spots, 16 spots were up-regulated and 17 down-regulated (Table 1).

On the basis of the previous analysis, preparative 2-D gels were loaded with 1 mg of protein lysate and run. The significant spots were excised with a manual spot picker and then submitted to trypsin in-gel digestion. The peptides recovered from the gel were subjected to nano-LC-MS/MS analysis, and the MS data were submitted to MASCOT database search to give the identification of 29 proteins (Table 1). Among them, several proteins were found in different isoforms or with different post-translational modifications and then detected in multiple spots, including actin (spots 1190, 1569, 1828, 2096) and vimentin (spots 541 and 542).

**Figure 3:**
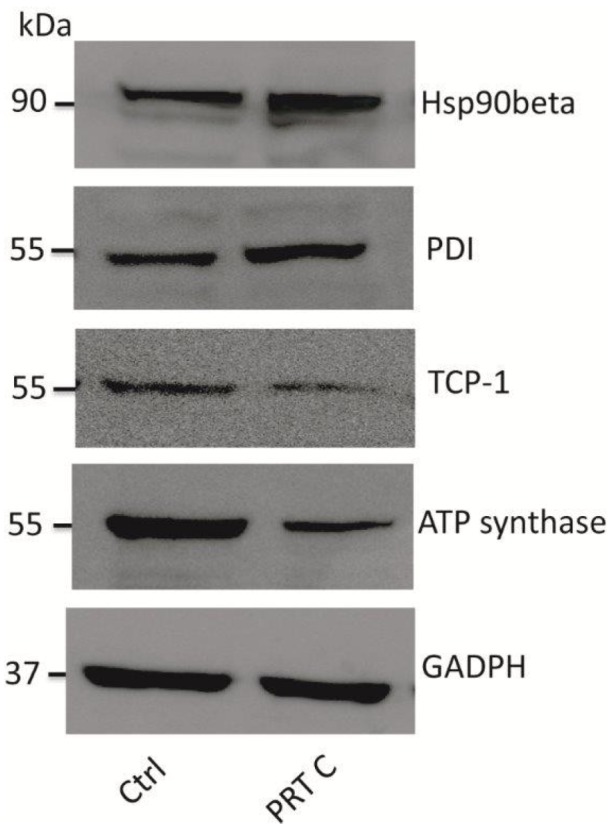
Western blotting of heat shock protein-90 (Hsp90), protein disulfide-isomerase A3 (PDI), T-complex protein 1 (TCP-1) and ATP synthase subunit β. Hsp90 and PDI levels increased while TCP-1 and ATP synthase subunit β levels decreased upon perthamide C incubation. GADPH is a standard control.

Western blotting *post hoc* validation was performed for the proteins Hsp90, protein disulfide-isomerase A3 (PDI), T-complex protein 1 (TCP-1) and ATP synthase subunit β. Immunoblot results were in total agreement with the DIGE data, confirming the up-regulation of Hsp90 and PDI and the down-regulation of TCP-1 and ATP synthase subunit-β (Figure 3).

**Figure 4:**
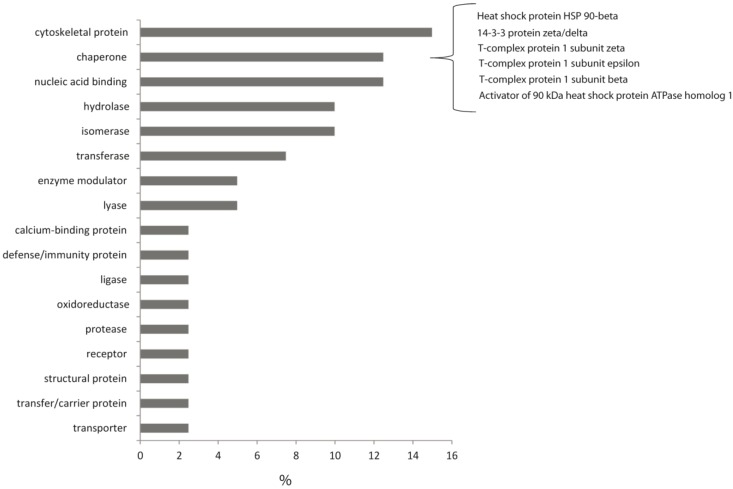
Structural annotation cluster analysis of differentially expressed proteins.

All the identified proteins were submitted to a functional annotation cluster analysis [17] to unravel their primary role in cell metabolism (Figure 4). They were grouped into several categories, most of them involved in the cytoskeleton formation (e.g., tubulin, actin isoforms, moesin, coronin, vimentin and gelsolin), while others in the folding processes (e.g., T-complex protein1 subunits ε, β, ζ, protein disulfide isomerase, heat shock protein 90 and its activator), in the nucleic acids binding (e.g., elongation factor, ribosomal proteins and heterogeneous nuclear ribonucleoproteins) and in the isomers rearrangement (phospho-glycerate mutase 1, triose-phosphate isomerase, hypoxanthine-guanine phospho-ribosyl-transferase).

**Table 1:** Proteins differentially expressed in J774 cells treated with perthamide C.

Spot			Proteins		Ratio	LC-MSMS		
	Accession No.	Mw (Da)	pI	Description		Coverage	Mascot Score	Peptide No.
1684	ALDOA_MOUSE	39787	5.29	Fructose-bisphosphate aldolase A	5.13	9%	120	6 (3)
*1752*	TPIS_MOUSE	27038	6.05	Triosephosphate isomerase	2.99	17%	334	9 (9)
920	HS90B_MOUSE	83615	5.88	Heat shock protein HSP 90-beta	2.95	7%	400	11 (9)
1086	TBB5_MOUSE	50095	8.97	Tubulin beta-5 chain	2.91	12%	240	14 (9)
1754	HPRT_MOUSE	24783	5.29	Hypoxanthine-guanine phosphoribosyl transferase	2.76	14%	152	8 (5)
*969*	MOES_MOUSE	67839	4.94	Moesin	2.76	8%	147	9 (4)
2096	ACTB_MOUSE	42052	6.67	Actin, cytoplasmic 1	2.64	36%	139	4 (3)
1965	1433Z_MOUSE	27925	6.90	14-3-3 protein zeta/delta	2.53	42%	163	8 (4)
1190	ACTB_MOUSE	42052	6.22	Actin, cytoplasmic 1	2.51	20%	321	12 (9)
1698	PGAM_MOUSE	28928	6.67	Phosphoglycerate mutase 1	2.27	20%	188	12 (7)
1828	ACTB_MOUSE	42052	4.73	Actin, cytoplasmic 1	2.24	25%	92	4 (3)
1569	ACTB_MOUSE	42052	4.97	Actin, cytoplasmic 1	2.22	15%	109	3 (3)
1533	PDIA3_MOUSE	57099	8.31	Protein disulfide-isomerase A3	2.13	19%	99	5 (2)
1542	ROA2_MOUSE	37437	6.21	Ribonucleoproteins A2/B1	2.08	44%	93	2 (2)
1058	COR1A_MOUSE	51641	4.78	Coronin-1A	2.02	33%	77	6 (3)
*337*	GELS_MOUSE	86287	8.02	Gelsolin	1.84	27%	200	8 (6)
676	LYZ1_MOUSE	17240	9.55	Lysozyme C-1	2.81	8%	41	1 (1)
692	ATPB_MOUSE	56265	5.19	ATP synthase subunit beta	2.72	42%	365	18 (16)
477	HNRPK_MOUSE	51230	5.39	Heterogeneous nuclear ribonucleoprotein K	2.64	19%	177	7 (6)
476	TCPZ_MOUSE	58424	5.72	T-complex protein 1-zeta	2.55	4%	68	5 (2)
454	TCPE_MOUSE	60042	7.23	T-complex protein 1-epsilon	2.43	4%	54	2 (1)
389	TKT_MOUSE	68272	7.27	Transketolase	2.41	10%	36	2 (1)
*541*	VIME_MOUSE	53712	5.91	Vimentin	2.31	28%	462	23 (16)
542	VIME_MOUSE	53712	5.91	Vimentin	2.18	40%	329	14 (12)
441	DPYL2_MOUSE	62638	4.83	Dihydropyrimidinase-related 2	2.17	16%	97	6 (4)
1117	TALDO_MOUSE	37534	6.37	Transaldolase	1.99	15%	72	5 (3)
561	TCPB_MOUSE	57783	6.31	T-complex protein 1 subunit beta	1.91	8%	171	4 (4)
1221	RLA0_MOUSE	34366	5.97	60S acidic ribosomal protein P0	1.90	13%	164	6 (5)
753	ENOA_MOUSE	47453	5.06	Alpha-enolase	1.86	29%	110	6 (4)
486	UBP14_MOUSE	56422	5.07	Ubiquitin carboxyl-terminal hydrolase	1.84	44%	63	1 (1)
1010	RSSA_MOUSE	32931	4.81	40S ribosomal protein SA	1.81	31%	228	9 (6)
966	AHSA1_MOUSE	38321	6.63	Activator of 90 kDa heat shock protein ATPase-1	1.81	10%	29	2 (1)
1336	ANXA5_MOUSE	35787	5.95	Annexin A5	1.63	13%	171	3 (3)

On the basis of our previous evidences on the involvement of perthamide C in the apoptosis [16], we focused our attention on the enzymes involved in the cellular folding processes. In particular, the expression of Hsp90, a main target of perthamide C [16], and 14-3-3 proteins increased, whereas T-complex protein 1 (subunit zeta, epsilon and beta) and Hsp90 activator expression was found to decrease. Indeed, these chaperones are required for the stability and activity of a range of client proteins playing a critical role in signal transduction, cellular trafficking, chromatin remodeling, cell growth, differentiation, and apoptosis [18]. Furthermore, the expression changes measured for Hsp90, 14-3-3 and T-complex proteins easily associate with an apoptosis reduction [19,20,21,22,23,24,25], and the anti-apoptotic role of perthamide C is also confirmed by the level alteration of other proteins, as annexin 5, dihydropyrimidinase-related protein 2 and gelsolin, independent from the folding process but in any case implicated in the apoptosis regulation [26,27,28].

ER stress modulates the apoptosis in a number of pathological processes, such as cancer and inflammation, and cytosolic and ER folding assistant proteins are considered emerging therapeutic targets [18,29]. All the data presented in this paper concur to the view that perthamide C effectively attenuates apoptosis [16], and shed more light on the cellular mechanisms of action of this marine potential lead.

## 3. Experimental Section

### 3.1. Cell Culture and Proteins Extraction

A cell line derived from murine macrophages J774.A1 was maintained in Dulbecco’s modified Eagle’s medium supplemented with 10% heat inactivated fetal bovine serum. The cells were cultured to 70% confluence and treated with 10 μM perthamide C and DMSO (control) in fresh media for 24 h. Cells were then scraped off and harvested by centrifugation (1000× *g*, 15 min, 4 °C) and washed twice with cold phosphate buffered saline (PBS). This is a routine lysis procedure which did not alter proteins’ expression levels [30].

Cell pellets were then re-suspended in 100 μL lysis buffer (30 mM Tris–HCl pH 8.8, 8 M urea, 2 M thiourea, 2% CHAPS and 2% DTT) together with a cocktail of protease inhibitors (Sigma Aldrich, St. Louis, MO, USA), and incubated on ice for 5 min. Samples were sonicated for two pulses of 10 s, at 30% output power, using a *Vibracell* sonicator. Soluble proteins were then collected in the surnatants upon a further centrifugation (10,000× *g*, 10 min, 4 °C) to remove insoluble parts. Proteins concentration was determined using the protein assay kit (BioRad, Hercules, CA, USA) according to the manufacturer’s instructions, using bovine serum albumin as a standard. Three biological replicates were collected.

### 3.2. Minimal CyDye Labeling

Proteins labeling was performed using the CyDyes DIGE Fluors developed for fluorescence 2-D DIGE technology according to the manufacturer’s recommended protocol (GE-Healthcare, Uppsala, Sweden). Briefly, 50 μg of each protein sample (5 μg/μL), were covalently labeled with a 400 pmol of CyDyes at 4 °C for 30 min in the dark. The internal standard was labeled with Cy2 while control and treated samples were labeled with Cy3 and Cy5, respectively. The reaction was quenched adding 1 μL of 10 mM lysine. The samples volume was adjusted to 30 μL using lysis buffer. Cy3- and Cy5-labeled protein samples as well as the Cy2-internal standard were mixed in 1:1:1 ratio. In the following step, labeled protein samples were diluted to 300 μL by the addition of 100 μL of destreak solution (GE Healthcare, Uppsala, Sweden) and 110 μL of rehydration buffer (7 M urea, 2 M thiourea, 4% CHAPS, 20 mg/mL DTT, 1% pharmalytes and trace amount of bromophenol blue) for the 18 cm immobilized pH gradient (IPG) strip.

### 3.3. Isoelectric Focusing (IEF) and SDS-PAGE

CyDyes labeled samples (150 μg protein in total) were loaded on 18-cm containing a pH gradient of 3–10 adding 1 mL of mineral oil on top. Proteins were focused using an Ettan IPGPhor 3 (GE Healthcare, Uppsala, Sweden) with the following settings: 300 V (3 h), 1000 V (6 h) and 8000 V (6 h). After IEF, the strips were equilibrated first in equilibration buffer (6 M urea, 30% glycerol, 2% SDS, 75 mM Tris–HCl buffer, pH 8.8, 0.01% bromophenol blue) supplemented with 2% DTT for 15 min and then with 2.5% iodoacetamide (in the same buffer) for 15 min. The IPG strips were then rinsed once in the SDS-gel running buffer before transfer onto the SDS-gel (12% SDS-gel prepared using low florescent glass plates) and sealed with 0.5% (w/v) agarose solution (in SDS-gel running buffer). The SDS-gels were run at 15 °C and until the dye front ran out of the gels. Electrophoresis was performed with 1-mm thick on a Ettan Dalt 12 System (GE Healthcare, Uppsala, Sweden).

### 3.4. Image Scan and Data Analysis

Image scans were carried out using the Ettan DIGE Imager (GE Healthcare, Uppsala, Sweden) according with manufacturer's protocols. In particular, Cy2 images were scanned using a 488 nm laser and a 520 nm/40 band-pass emission filter. Cy3 images were scanned using a 532 nm laser and a 580 nm/30 BP emission filter, while Cy5 images were scanned using a 633 nm laser and a 670 nm/30 BP emission filter. Gel analysis was performed using ImageMaster 2D Platinum (GE Healthcare Bio-sciences, Uppsala, Sweden) to co-detect and determine the relative quantification of spot intensities.

The biological variation analysis module was then used to match protein spots among different gels and to identify protein spots that exhibited statistically significant differences. Paired t-test analysis was performed for every matched spot set and protein spots showing a significant (*p* < 0.05) quantitative difference between samples with abundance change of at least 1.6 times were manually assigned as “spot of interest”.

### 3.5. Spot Picking and Digestion

Preparative 2D-DIGE gels were stained with Comassie G-250 (BioRad, Hercules, CA, USA) overnight and fixed in 40% methanol/10% acetic acid. Preparative gel images were matched to those of the DIGE gels, and the spots of interest were manually excised. The gel slices were transferred into a 500 μL microcentrifuge tube, treated with 100 μL ammonium bicarbonate (50 mM) supplemented with 50% acetonitrile and incubated for 15 min. The above step was repeated twice. The slices were washed twice with 100 μL water for 5 min and then soaked in 100% acetonitrile for 15 min. Acetonitrile was removed and then the gels were dried in vacuum for 10 min at room temperature for 30 min. Gel pieces were subsequently rehydrated with appropriate volume of trypsin solution (10 μg/mL in 25 mM ammonium bicarbonate) to wet the entire gel surface. After 60 min at 4 °C, following the addition of 30 µL ammonium bicarbonate (25 mM), samples were digested overnight at 37 °C. The supernatants were collected and peptides were extracted from the gel slices using 100% CH_3_CN. Finally, the supernatant was collected and both were combined. All peptide samples were dried out and dissolved in 10% FA before mass spectrometry analysis.

### 3.6. MS Analysis and Database Searching

Peptide mixture (5 μL) were injected onto a nano Acquity LC system (Waters Corp., Manchester, UK). The peptides were separated on a 1.7 μm BEH C-18 column (Waters Corp., Manchester, UK) at a flow rate of 1000 nL/min. The gradient (Solution A: 0.1% formic acid, solution B: 0.1% formic acid, 100% ACN) started at 5% and ended at 50% B after 55 min. MS and MS/MS data were acquired using a Q-TOF Premier mass spectrometer (Waters Corp., Micromass, Manchester, UK). Doubly and triply charged peptide-ions were automatically chosen by the MassLynx software and fragmented. MS data were automatically processed and peak-lists for protein identifications by database searches were generated by the ProteinLynx software. Database searches were carried out with MASCOT server using the SwissProt protein database. The SwissProt human database (release 2010_11 of 2 November 2010, 522,019 sequences, 184,241,293 residues) was searched allowing 2 missed cleavages, carbamidomethyl (C) as fixed modification, oxidation (M) and phosphorylation (ST) as variable modifications. The peptide tolerance was set to 80 ppm and the MS/MS tolerance to 0.6 Da.

### 3.7. Immunoblotting

*Post hoc* validation of proteomic data was performed using Western immuno-blotting analysis. Protein concentration was determined using Bradford assay and bovine serum albumin (BSA) was used as standard. Proteins (30 μg) for control and treated samples were resolved on 12% denaturing polyacrylamide gel and transferred onto a nitrocellulose membrane. Membranes were incubated for 1 h at RT in a blocking solution (25 mm Tris (pH 8), 125 mm NaCl, 0.1% Tween-20, 5% nonfat dried milk) and then for 16 h at 4 °C with anti-Hsp90 (BD Biosciences, Franklin Lakes, NJ, USA), anti-PDI (BD Biosciences, Franklin Lakes, NJ, USA), anti-TCP-1 (Santa Cruz Biotechnology, Santa Cruz, CA, USA) and anti-ATP synthase subunit β (Sigma-Aldrich, St. Louis, MO, USA) antibodies at 1:1000 dilution of each primary antibody. Finally, membranes were incubated for 1 h with a peroxidase-conjugated secondary antibody (1:5000, ThermoFisher, Waltham, MA, USA). Proteins were detected by the enhanced chemiluminescence (ECL) detection system LAS 4000 (GE Healthcare, Waukesha, WI, USA). As a control for equal protein loading, glyceraldehyde-3-phosphate dehydrogenase (GAPDH) was used. Membranes were probed for GAPDH using a monoclonal antibody (1:1000 dilution, Santa Cruz Biotechnology, Santa Cruz, CA, USA). The immuno-blotting detection was performed as reported above.

## 4. Conclusions

The chemical investigation of marine organisms has a huge biomedical potential and marine derived compounds represent an essential component of the current research arsenal aimed to the discovery of novel therapeutic targets. In this context, the elucidation of all cellular pathways affected by natural compounds is crucial to point out their biological action and possible side effects. This task can be achieved through the differential analysis of a cellular proteome after treatment with the natural product.

In this paper we investigated the effect of perthamide C—a novel cyclic octapeptide isolated from the polar extract of *Theonella swinhoei* and endowed with a broad and interesting anti-inflammatory profile—on the proteome of J774 murine macrophage cells by two-dimensional DIGE. Perthamide C modulates the expression of several cytosolic and ER-associated proteins, mainly involved in the folding processes, and strictly linked to the apoptotic signaling. Therefore, these data shed light on the cellular pathways altered by perthamide C and suggest its possible role in the reduction of the apoptosis process, providing new insights into its biological effects.
